# *Helicobacter suis*-Associated Gastritis Mimicking Conventional *H. pylori*-Associated Atrophic Gastritis

**DOI:** 10.1155/2022/4254605

**Published:** 2022-07-20

**Authors:** Masaya Iwamuro, Somay Yamagata Murayama, Masahiko Nakamura, Kenta Hamada, Takehiro Tanaka, Hiroyuki Okada

**Affiliations:** ^1^Department of Gastroenterology and Hepatology, Okayama University Graduate School of Medicine, Dentistry and Pharmaceutical Sciences, Okayama 700-8558, Japan; ^2^Department of Fungal Infection, National Institute of Infectious Diseases, Tokyo 162-8340, Japan; ^3^Omura Satoshi Memorial Institute, Kitasato University, Tokyo 108-8641, Japan; ^4^Department of Pathology, Okayama University Graduate School of Medicine, Dentistry and Pharmaceutical Sciences, Okayama 700-8558, Japan

## Abstract

A 45-year-old Japanese man underwent esophagogastroduodenoscopy, which revealed spotty redness at the gastric fornix, mucosal swelling, diffuse redness in the corpus, and mucosal atrophy in the gastric angle and antrum. Histological examination showed rod-shaped bacteria that appeared larger than *Helicobacter pylori*. The patient tested positive for rapid urease test, and serum anti-*H. pylori* IgG antibody test results were negative. Further examination of the bacteria revealed that *H. suis* antibody test was positive, and the presence of *H. suis* was confirmed using *H. suis*-specific real-time PCR. *H. suis* was successfully eradicated after triple therapy with vonoprazan, amoxicillin, and clarithromycin. This case reinforces the notion that non-*H. pylori* Helicobacter species such as *H. suis* and *H. heilmannii* may be involved in the pathogenesis of active gastritis in patients who test negative for *H. pylori* antibodies.

## 1. Introduction

Since its discovery in 1982, *Helicobacter pylori* has been widely recognized as a pathogenic organism that causes gastritis, peptic ulcer disease, and gastric cancer [1]. In addition to *H. pylori*, various spiral-shaped organisms have been identified in humans and other animals. Helicobacter species other than *H. pylori* are known as non-*Helicobacter pylori* Helicobacter, which include *H. suis*, *H. heilmannii*, *H. bizzozeroni*, *H. salomonis*, and *H. felis*. Among these, *H. suis* is the primary human pathogenic organism [2].

Although *H. suis* infection in the human stomach causes gastritis, the clinicopathological features of *H. suis*-associated gastritis have long been uninvestigated because its isolation and cultivation are technically difficult [3]. Herein, we report the case of a patient with *H. suis*-associated gastritis, who showed endoscopic features similar to those of conventional *H. pylori*-associated atrophic gastritis.

## 2. Case Presentation

**Figure 1:**
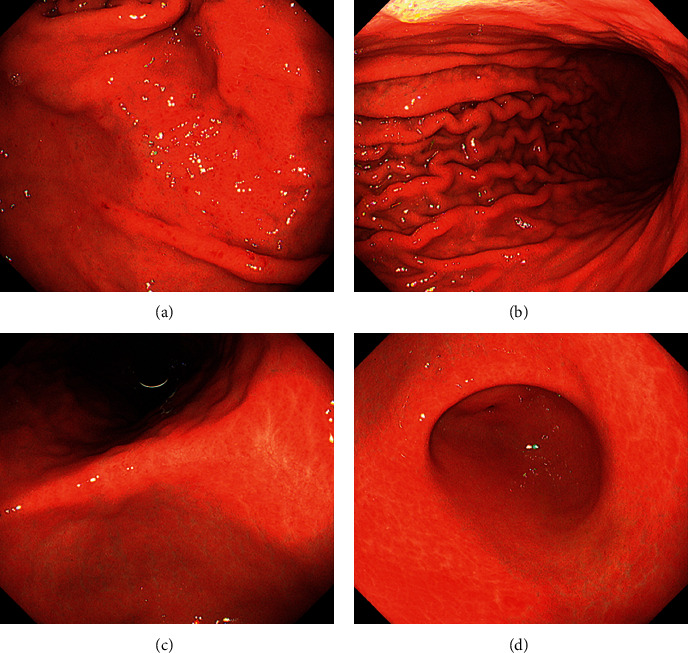
Esophagogastroduodenoscopy images. Endoscopy reveals spotty redness at the gastric fornix (a), mucosal swelling with diffuse redness in the corpus (b), and mucosal atrophy in the gastric angle (c) and antrum (d).

**Figure 2:**
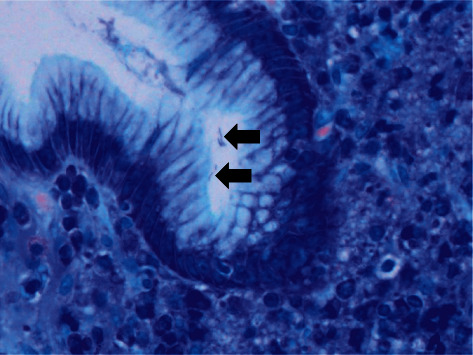
Histological image. Giemsa stain shows rod-shaped bacteria appearing larger than *H*. *pylori* (arrows), suggesting a non-*Helicobacter pylori* Helicobacter infection.

A 45-year-old Japanese male underwent upper gastrointestinal series and was diagnosed with chronic gastritis. Subsequently, the patient underwent esophagogastroduodenoscopy, which revealed spotty redness at the gastric fornix (Figure 1(a)), mucosal swelling, diffuse redness in the corpus (Figure 1(b)), and mucosal atrophy at the gastric angle (Figure 1(c)) and antrum (Figure 1(d)). Regular arrangement of collecting venules was scarcely identified throughout the stomach. Thus, atrophic gastritis and current infection of *H. pylori* were suspected. However, the serum anti-*H. pylori* IgG antibody test results were negative. Histological examination revealed rod-shaped bacteria that appeared larger than *H. pylori* (Figure 2, arrows). Infection of non-*H. pylori* Helicobacter species, such as *H. suis* and *H. heilmannii*, was then suspected, and the patient was referred to our hospital for further investigation.

**Figure 3:**
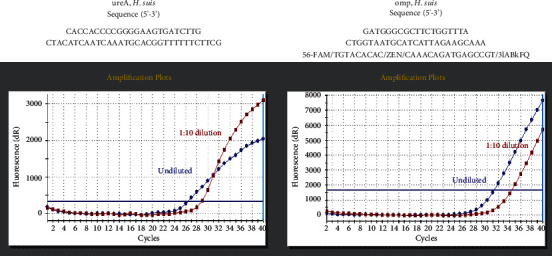
PCR analysis. The presence of *H*. *suis* is confirmed using *H*. *suis*-specific real-time PCR, targeting ureA and outer membrane protein genes.

**Figure 4:**
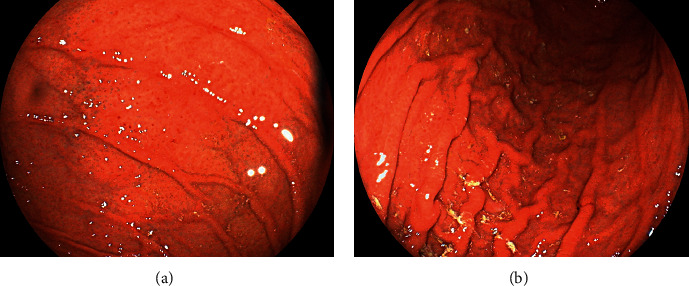
Esophagogastroduodenoscopy images two months after the eradication therapy. Spotty redness still exists at the gastric fornix (a), while mucosal swelling and diffuse redness are not observed in the corpus (b).

The patient had no history of other diseases and was not taking any medication. Physical examination revealed no abnormalities and no evidence of hepatosplenomegaly or peripheral lymphadenopathy. All laboratory findings, including lactate dehydrogenase and soluble interleukin-2 receptor levels, were within the normal ranges. Repeat esophagogastroduodenoscopy and biopsy showed rod-shaped bacteria that seemed larger than *H. pylori*. The patient tested positive in the rapid urease test. The serum anti-*H. pylori* IgG antibody test was negative, whereas the in-house ELISA test for the *H. suis* antibody was positive. The presence of *H. suis* was further confirmed using *H. suis*-specific real-time polymerase chain reaction (PCR) targeting ureA and outer membrane protein genes (Figure 3) [4, 5]. *H. pylori* was negative on PCR test. Consequently, the patient was diagnosed with *H. suis*-associated gastritis. *H. suis* was successfully eradicated after triple therapy with vonoprazan, amoxicillin, and clarithromycin. Esophagogastroduodenoscopy performed two months after eradication therapy showed that spotty redness remained at the gastric fornix (Figure 4(a)), while mucosal swelling and diffuse redness in the corpus disappeared (Figure 4(b)).

## 3. Discussion

*H. suis* is a zoonotic pathogen that colonizes the gastric mucosa of 60–95% of pigs at the slaughter age. *H. suis* primarily infects the fundic and pyloric gland zones of the porcine stomach, leading to gastritis, gastric ulcers, and decreased daily weight gain [6]. Since the first human case of *H. suis*-associated erosive gastritis was reported in 1994 [7], *H. suis* infection in humans has been described in only a small number of cases. This is mainly because it is difficult to isolate and cultivate the pathogen. Rimbara et al. reported that *H. suis* is not viable at neutral pH. Thus, endoscopic biopsy specimens containing *H. suis* require a low-pH medium for transport and successful isolation [3]. In the present patient, an in-house enzyme-linked immunosorbent assay for the detection of human IgG antibodies to *H. suis* was positive. In addition, we extracted the DNA from the gastric biopsy specimens and performed nested PCR using the specific primers for *H. suis* [8], which was also positive. Consequently, the patient was diagnosed with *H. suis*-associated gastritis.

Although *H. suis* has urease activity, its activity is generally lower than that of *H. pylori* [9]. Therefore, the urea breath test and rapid urease test, which are commonly used to detect urease activity in *H. pylori*, often yield negative results for *H. suis* [3, 10]. However, the patient tested positive in the rapid urease test. Since both serum *H. pylori* antibody and PCR assays for *H. pylori* were negative, co-infection with *H. suis* and *H. pylori* was unlikely. Consequently, the urease activity of *H. suis* detected in the present patient may have been comparable to that of *H. pylori*. Another explanation is that the bacterial density of *H. suis* was greater than that of other patients with *H. suis* infections.

The typical endoscopic features of non-*Helicobacter pylori* Helicobacter-associated gastric lesions include spotty redness, crack-like mucosa, nodular gastritis-like appearance, and white marbled appearance. Erosions, ulcers, and even extranodal marginal zone lymphoma of the mucosa-associated lymphoid tissue can be found in the stomach, in association with non-*Helicobacter pylori* Helicobacter infection [11, 12]. Tsukadaira et al. investigated 50 patients with non-*Helicobacter pylori* Helicobacter infections in the stomach, including 26 cases of *H. suis* and two cases of *H. heilmannii*/*H. ailurogastricus* [13]. The authors reported that the crack-like mucosa was observed in 45 out of 50 patients (90.0%), which was defined as a mesh-like morphology composed of faded, depressed, and varying width lines on coarse and slightly reddish mucosa extending from the gastric antrum to the angle. Nodular gastritis was noted in 11 out of 50 patients (22.0%). All the patients had a regular arrangement of collecting venules in the gastric corpus (100%). Shiratori et al. reported two patients with non-*Helicobacter pylori* Helicobacter infection of the stomach [14]. Both patients had a white marble appearance in the lesser curvature of the gastric angle and antrum, and the authors speculated that the white marble appearance is a potential characteristic finding of non-*Helicobacter pylori* Helicobacter infection gastritis.

Table 1 shows the endoscopic features of the stomach that are characteristic of patients with current *H. pylori* infection according to the Kyoto classification of gastritis [15]. They include atrophy, diffuse redness, foveolar-hyperplastic polyps, xanthomas, intestinal metaplasia, mucosal swelling, patchy redness, depressive erosions, enlarged and tortuous folds, sticky mucus, spotty redness, and nodularity. In contrast, fundic gland polyps and the regular arrangement of collecting venules are absent or reduced in the stomachs of patients with current *H. pylori* infection, as opposed to those of people without the infection. Map-like redness is a representative endoscopic feature observed in the stomachs of patients in whom *H. pylori* infection has been previously eradicated.

The esophagogastroduodenoscopy performed in the present patient revealed that the crack-like mucosa and white marble appearance were absent, and a regular arrangement of collecting venules was scarcely identified throughout the stomach. Instead, spotty redness at the gastric fornix, mucosal swelling, diffuse redness in the corpus, and mucosal atrophy in the gastric angle were observed (Table 1). In a previous report, the spotty redness in the corpus was found in 4 out of 50 patients (8.0%), and mucosal swelling was observed in only 1 out of 50 patients (2.0%) [13]. The endoscopic features of our patient were indistinguishable from those of conventional *H. pylori*-induced gastritis. Although the exact cause of the infrequent endoscopic appearance of *H. suis*-associated gastritis remains unclear, the higher urease activity, which was presumed from the positive results of the rapid urease test, might be involved in the unique endoscopic features of the present patient. However, as this report describes only one patient, this issue must be investigated.

## 4. Conclusions

This case reinforces the notion that non-*H. pylori* Helicobacter species, such as *H. suis,* may be involved in the pathogenesis of active gastritis in patients who tested negative for *H. pylori* antibodies. It is also noteworthy that *H. suis*-associated gastritis may present with endoscopic features indistinguishable from those of *H. pylori* infection.

## Figures and Tables

**Table 1:** Endoscopic features according to the Kyoto classification of gastritis.

	Typical case of active infection with *H. pylori*	Present patient
Entire stomach		
Atrophy	Present	Present
Diffuse redness	Present	Present
Foveolar-hyperplastic polyp	Present	Absent
Xanthoma	Present	Absent
Intestinal metaplasia	Present	Absent
Mucosal swelling	Present	Present
Patchy redness	Present	Absent
Depressive erosion	Present	Absent
Map-like redness	Absent	Absent

Gastric body		
Enlarged and tortuous folds	Present	Present
Sticky mucus	Present	Absent

Gastric fornix and body		
Spotty redness	Present	Present
Fundic gland polyp	Absent	Absent

Lower gastric body to antrum		
Nodularity	Present	Absent
Regular arrangement of collecting venules	Absent	Absent

## Data Availability

The data used to support the findings of this study are restricted in order to protect patient privacy.
